# Analysis of the Effects of Post-Fermentation Freezing Treatment on the Flavor Characteristics of Beibinghong Ice Wine by HPLC and HS-GC-IMS

**DOI:** 10.3390/foods14091631

**Published:** 2025-05-06

**Authors:** Yining Sun, Pengqiang Yuan, Guoliang Liu, Yiming Yang, Nan Shu, Wenpeng Lu

**Affiliations:** Institute of Special Animal and Plant Sciences, Chinese Academy of Agricultural Sciences, Changchun 130112, China; 82101225210@caas.cn (Y.S.); 82101222242@caas.cn (P.Y.); 82101232245@caas.cn (G.L.); yangyiming@caas.cn (Y.Y.); shunan@caas.cn (N.S.)

**Keywords:** ice wine, freezing treatment, HPLC, HS-GC-IMS, sensory evaluation

## Abstract

This study aimed to investigate the effects of post-fermentation freezing treatment on the flavor characteristics of Beibinghong ice wine. Physicochemical indices, organic acids, volatile compound content, odor activity values (OAVs), and sensory attributes of ice wines subjected to different treatments were systematically analyzed using high-performance liquid chromatography (HPLC), headspace gas chromatography–ion mobility spectrometry (HS-GC-IMS), sensory evaluation, and orthogonal partial least squares discriminant analysis (OPLS-DA). The results demonstrated significant differences in fundamental physicochemical indices between freezing-treated and control samples. The freezing treatment significantly reduced total acid, total sugar, and tannin content, thereby alleviating cloyingly sweet and pungent taste sensations and achieving a more harmonious and balanced flavor profile. Concurrently, alterations in the levels of esters, alcohols, and other volatile compounds were observed, with 10% alcohol-by-volume (ABV) freezing-treated samples exhibiting optimal performance in aromatic complexity (featuring fruity and honey notes) and taste balance. Sensory evaluation further confirmed that freezing treatment enhanced the delicacy and complexity of ice wine aromas. This study demonstrates that post-fermentation freezing treatment effectively optimizes the flavor profile of ice wine, providing a theoretical foundation for refining ice wine production processes.

## 1. Introduction

*Vitis amurensis* Rupr. is a deciduous vine plant, grape genus *Vitis*, native to northeastern China, the Russian Far East, and the Korean Peninsula. Mainly distributed in northeastern China, Inner Mongolia, etc., is one of the most cold-resistant species in the genus *Vitis* [1]. The Institute of Special Animal and Plant Sciences of the Chinese Academy of Agricultural Sciences has developed premium wine cultivars including Beibinghong , Beiguohong, Shuanghong, Xuelanhong, and Zuoyouhong [2], renowned for their exceptional frost tolerance and disease resistance. Wines produced from these cultivars exhibit robust textures and multilayered aromatic complexity, combining functional resilience with organoleptic superiority, thereby securing their prominence in viticulture. The Beibinghong is the world’s first wild grape variety to produce ice wine [3]. Beibinghong ice wine is a sweet wine made from the juice of naturally frozen grapes on the vines when the temperature drops to −7 to ~−8 °C. Ice wine has a deep ruby-red color, rich and pleasant aroma of honey and almonds, and prominent fruity aroma, which is a unique style of ice wine, and is very popular in the northeast of China [4]. However, changing market demands have shifted the focus of wine from yield to quality, and chemical and sensory attributes have become key benchmarks for evaluating the quality of ice wine.

Organic acids are key chemical parameters that directly affect the quality of wine through their influence on flavor profile, chemical stability, and pH regulation [5]. Nowadays, advances in chromatographic techniques have improved the detection of organic acids, and high-performance liquid chromatography (HPLC) has been widely used because of its rapidity, simplicity, and high sensitivity [6].

Aroma, an important sensory attribute of wine, relies on the analysis of volatile components. The main detection methods include comprehensive two-dimensional gas chromatography (GC × GC), gas chromatography–mass spectrometry (GC-MS), gas chromatography–ion mobility spectrometry (GC-IMS), and electronic nose system (E-nose). GC-IMS, a novel analytical platform combining gas-phase separation with ion mobility detection, operates at atmospheric pressure without requiring vacuum systems or extensive sample pretreatment. Its advantages include rapid response, simple operation, and real-time detection of volatile/semi-volatile organic compounds. In contrast, MS necessitates high-vacuum environments, while GC × GC—though suited to complex matrices—is less routinely applied than GC-MS [7].

Cold treatment processes have become a key research direction in winemaking technology in recent years due to their potential benefits for wine quality and uniqueness [8]. For example, under natural freezing conditions (such as the freeze–thaw process of grapes on the vine), juice concentration triggers a series of chemical reactions, significantly altering the concentrations of volatile phenols, lactones, and characteristic aldehydes [9]. Artificial cold treatment processes (e.g., pre-fermentation freezing techniques) enhance the release of aromatic compounds by increasing intracellular liquid volume, thereby influencing the aroma profile of wines [10]. Studies also indicate that freezing treatments promote the release of anthocyanins, tannins, and aroma precursors, with lower treatment temperatures yielding better effects [11]. Existing research has predominantly focused on the impacts of pre-fermentation freezing [12,13,14], while studies investigating post-fermentation freezing treatment remain limited. Therefore, clarifying whether post-fermentation freezing treatment affects wine chemical composition or improves the sensory quality of ice wine has become a critical point for optimizing ice wine production theory.

In this study, ice wine was fermented from Beibinghong *Vitis amurensis* Rupr., detected by GC-IMS and HPLC, and the flavor components of ice wine were analyzed by VOCal software (version 0.4.03), combined with orthogonal partial least squares discriminant analysis (OPLS-DA) to screen for discriminant compounds with VIP values > 1 potential, and odor activity values (OAVs) were calculated to identify key volatile organic compounds (VOCs) in the different treated ice wine samples. Total acids, total sugars, total phenols, anthocyanins, tannins, pH, organic acids, volatile compounds, and sensory evaluation were used as the evaluation indices to analyze the quality differences between different treatments of post-fermentation wine samples in order to elucidate the effects of post-fermentation freezing treatments on the chemical profiles of wines.

## 2. Materials and Methods

### 2.1. Materials

The experimental material consisted of Beibinghong ice grapes harvested in December 2023 from the experimental vineyard of the Institute of Special Animal and Plant Sciences, Chinese Academy of Agricultural Sciences (Ji’an City, Jilin Province, China). Grapes were collected at the overripe stage of berry development, and 80 kg was randomly sampled for ice wine production.

### 2.2. Experimental Methods

#### 2.2.1. Winemaking Process

**Figure 1:**
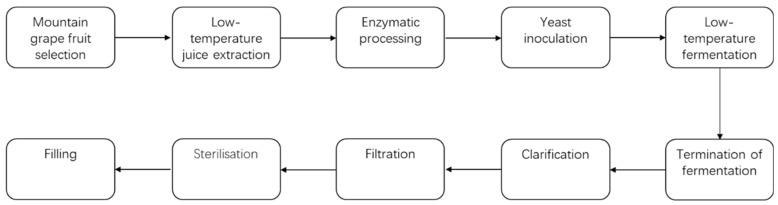
Ice wine fermenting process flowchart.

The same grape juice was used in the fermentation process, and the specific physicochemical parameters are shown in Table 1. A process flowchart for ice wine fermenting is shown in Figure 1.

Key Operational Steps:

(1) Harvesting: Two months after the normal harvesting period (around December 5), the grapes are harvested when the temperature was below −7 °C, ensuring that the berries were completely frozen.

(2) Selection of Frozen Berries: Berries with uniform maturity and free from pests, diseases, or spoilage were selected for processing.

(3) Low-Temperature Pressing: Manual pressing was conducted while berries remained frozen. Potassium metabisulfite (0.0525 g/L) and pectolytic enzyme VR-L (0.05 mL/kg) were added. The temperature was adjusted to 15 °C and allowed to settle for 24 h.

(4) Yeast inoculation: BV818 was activate at a ratio of 1:10 (yeast–water) in warm water (38–40 °C) containing 1/3 juice and 2/3 distilled water.

Activation was confirmed by vigorous foaming, and the yeast slurry was then added to the grape juice.

(5) Low-Temperature Fermentation: Fermentation lasted approximately 50 days at 15–18 °C.

(6) Daily Monitoring: Alcohol content was measured daily using a hydrometer.

Samples were collected at each 1% vol. increment once alcohol reached 9% vol., continuing until fermentation ceased (total of three samples between 9% and 11% vol.).

(7) Sample Treatment.

Test Group: Wines were stored at −20 °C for one month.

Control Group: Wines were stored at 16 °C.

All samples underwent thermal pasteurization at 80 °C for 2 min prior to storage [15]. Post-treatment, wines were subjected to quality testing and analysis. Sample numbers are shown in Table 2.

#### 2.2.2. Determination of Basic Physicochemical Parameters in Beibinghong Ice Wine

Alcohol content and total acidity were measured according to GB/T 15038-2006 (Analytical methods of wine and fruit wine) [16]. Total sugar content was determined using the anthrone–sulfuric acid colorimetric method, with a standard curve prepared from glucose solutions. Tannin content was analyzed via the Folin–Denis method, and a standard curve was established for different tannin concentrations. Total phenolic content was quantified using the Folin–Ciocâlteu method [17], and total anthocyanin content was measured following the pH differential method [18].

#### 2.2.3. Determination of Organic Acid Content in Beibinghong Ice Wine

The experiment was carried out using Agilent 1200 series HPLC system (Agilent Technologies Ltd., Santa Clara, CA, USA). Quantitative analysis of organic acids in ice wine was conducted using high-performance liquid chromatography (HPLC) with the following parameters. An Agilent C18-XT column (250 mm × 4.6 mm i.d., 5 μm particle size) was used at a temperature of 25 °C. The mobile phase consisted of phosphoric acid aqueous solution (pH 2.3) and methanol at a ratio of 97:3 (*v*/*v*) at a flow rate of 0.4 mL min^−1^. The wavelength of UV detection was 210 nm, and the injection volume was 10 μL [19]. The standard curves of the six organic acids measured are shown in Supplementary Materials Table S1.

#### 2.2.4. Headspace Gas Chromatography–Ion Mobility Spectrometry (HS-GC-IMS) Analysis

Experiments were performed using a FlavourSpec^®^ flavor analyzer (Shandong Haineng Scientific Instrument Co., Ltd., Zibo, China). Volatile compounds in ice wine were detected by GC-IMS using an automated headspace injection method. The assay procedure was as follows:

A wine sample (1 mL) was placed in a 20 mL headspace sample vial, 20 µL of 10 mg/L 4-methyl-2-pentanol (internal standard, IS) was added, the vial was sealed, and three replicate samples were prepared. The samples were incubated for 10 min at 60 °C with stirring at 500 rpm [20]. The headspace injection volume was 100 μL, the injector temperature was 85 °C, the column was an MXT-WAX column (30 m × 0.53 mm, 1 μm film thickness) with a temperature of 60 °C, the IMS detector temperature was 45 °C, the drift and carrier gases were nitrogen with a drift gas rate of 150 mL The carrier gas flow rate was started at 2 mL⋅min^−1^, held for 2 min, increased linearly to 10 mL⋅min^−1^ in the next 8 min, the flow rate was increased to 100 mL/min at 20 min and maintained at this rate for the remaining 10 min of the experiment. The total analysis time was 30 min.

#### 2.2.5. Relative Quantification of Volatile Compounds and Calculation of OAV of Samples

Peak volume integration of volatile organic compounds was assessed using the VOCal plug-in, and relative quantification was performed by the internal standard method. The formula is given below:

$$Ci=\frac{Cis*Ai}{Ais}$$

where *Ci* denotes the calculated mass concentration of the volatile organic compound (μg·L^−1^), *Cis* denotes the mass concentration of the internal standard (4-methyl-2-pentanol, μg·L^−1^), *Ai* corresponds to the peak volume of the compound, and *Ais* denotes the peak volume of the internal standard. The internal standard (4-methyl-2-pentanol) was formulated at a concentration of 198 μg·L^−1^ with a peak volume of 560.70, and the intensity of each peak was approximately equal to 0.353 μg·L^−1^

Based on the relative quantification of volatile organic compounds, the OAV was calculated as:

$$OAV=\frac{Ci}{OTi}$$

where *Ci* is the compound concentration (μg·L^−1^) and *OTi* represents the odor threshold (μg·L^−1^) obtained from the literature. Compounds with OAV > 1 are considered to contribute directly to the overall aroma profile of ice wine, while the contribution of compounds with OAV < 1 is minimal. A higher OAV value indicates a more intense aroma.

### 2.3. Quantitative Descriptive Sensory Analysis

Ice wine was evaluated by quantitative descriptive sensory analysis [21]. The analysis was conducted by 12 people (6 males and 6 females, aged 24–55 years) trained in observing and tasting taste aromas, according to the international standard ISO 11035 [22]. Ethics approval was obtained from the Institute of Special Animal and Plant Sciences of Chinese Academy of Agricultural Sciences, and informed consent was obtained from all subjects involved in the study. Each group of wine samples was taken (50 mL) each and placed in standard wine tasting glasses at a temperature of 18 °C. The tasting room was properly lit and free of any odor. Each sample was assigned a 3-digit number and presented to the judges in a randomized order, then scored according to a sensory evaluation scale, with specific evaluation of taste and aroma. The taste evaluation scale is shown in Supplementary Materials Table S2. The aroma evaluation table is shown in Supplementary Materials Table S3. The aroma of the mountain grape ice wine was scored by descriptive words (floral, fruity, botanical and herbaceous, fermented, tarry, and confectionery), and the corresponding aroma intensity was rated and scored on a 9-point scale, with ratings in the range of 0–3 for low intensity, 3–6 for medium intensity, 6–9 for high intensity. Each sample was evaluated in triplicate. The sensory evaluation form is detailed in Table 3.

### 2.4. Data Processing

Excel 2016 was used to statistically organize the experimental data, and analysis of variance (ANOVA) was performed using SPSS version 27.0 software. Statistical analysis of variance was performed on the experimental data to check for significant differences in each result, and the data are all expressed as means ± standard deviation, with *p* < 0.05, which was considered to be a significant difference between the two groups. Histograms were plotted using Origin 2021. OPLS-DA and VIP values were analyzed using Simca 17.0 software. Volatile organic compounds detected by HS-GC-IMS were analyzed by VOCal software (version 0.4.03). Differences in spectra were compared between samples using the Reporter plug-in. The Gallery Plot plug-in was used to compare fingerprinting, and characterization was performed using the Library Search plug-in. Clustered heatmaps were plotted using TBtools (version 2) and radar plots and dot-bar heatmaps were plotted by an online cloud tool platform (https://cloud.metware.cn/#/home (accessed on 20 November 2024)).

## 3. Results and Analysis

### 3.1. Physicochemical Characteristics of Beibinghong Ice Wines Under Post-Fermentation Treatments

**Figure 2:**
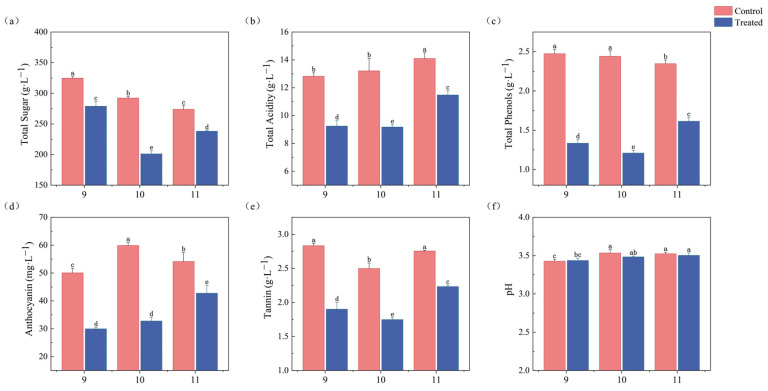
Changes in physicochemical indices of ice wine treated at 16 °C control and −20 °C. (**a**) Total sugar. (**b**) Total acid (expressed as tartaric acid). (**c**) Total phenols (expressed as gallic acid equivalents). (**d**) Anthocyanin. (**e**) Tannins (expressed as tannic acid equivalents). (**f**) pH. Different lowercase letters indicate significant differences between treatments. (Duncan’s test *p* < 0.05).

As shown in Figure 2, the physical and chemical indices of the ice wine samples treated with freezing after fermentation differed significantly from those of the control group (*p* < 0.05). Six ice wine samples had an alcohol content between 9% and 11% vol, which was in accordance with the requirements of Chinese national standard GB/T 25504-2010 Icewines (alcohol content: 9%–14% vol) [23]. The freezing treatment had a significant effect on total sugar, total acidity, total phenols, anthocyanins, and tannins, while the pH value changed little.

According to GB/T 25504-2010 Icewines, the total sugar content must exceed 125 g/L. The total sugar content of all the samples ranged from 201.09 to 324.60 g/L. The total sugar content of the samples treated by freezing (three groups) was lower than that of the control group, which improved the sweet and creamy taste of the ice wine. Total acidity was reduced by 27.90% (9T vs. 9C), 30.54% (10T vs. 10C), and 18.55% (11T vs. 11C) in the freeze-treated groups compared to their respective controls, with the greatest reduction occurring in the 10% alcohol group.

The total phenolic content was in the order of 9C > 10C > 11C > 11T > 9T > 10T. The anthocyanins, which are essential for color stability and health promotion [24], were highest in the freeze-treated 11T at 42.75 mg/L, while the control 10C had the highest anthocyanin content of 59.89 mg/L. The total phenolic content of the wines in the control 10C was also higher than that of 11T. Tannin content affects the astringency, antioxidant properties, and sense of structure of wines [25]. The tannin content of the freeze-treated samples was reduced, with the lowest level being 1.75 g·L^−1^. The reduction in tannin content reduced the astringency of the ice wines, and resulted in a more harmonized and balanced flavor of the ice wines.

### 3.2. Organic Acid Profiles of Beibinghong Ice Wines Under Post-Fermentation Treatments

The second-most important chemicals in wine after sugar are the organic acids, which are mainly composed of tartaric, malic, citric, and lactic acids [26]. Malic, tartaric, and citric acids are produced in the grape juice, while succinic, lactic, acetic, and fumaric acids are mainly produced during the winemaking process [27]. Tartaric acid has a strong acidic odor and is one of the major organic acids [28].

Among the ice wine samples, 9C had the highest tartaric acid content of 2.90 g/L (*p* < 0.05), followed by 10C, while 10T had the lowest tartaric acid content of 1.40 g/L. Malic acid is also one of the key characteristic organic acids in wine [29], and the malic acid content was 9C > 10C > 11C > 11T > 9T > 10T, with the lowest content of 10T at 4.70 g/L, which was significantly lower than the other five samples (*p* < 0.05). In addition to the fermentation process, lactic acid is also produced during the end period of alcoholic fermentation, and has a softer and more delicious flavor than malic acid [30]. Lactic acid content was the highest in 11C, 1.19 g/L, and the lowest in 10T, 0.53 g/L. Citric acid has a refreshing taste and can enrich the mouthfeel of the wines [31], with the highest content in 9C, 0.75 g/L, followed by 11C, and the lowest was 10T, 0.42 g/L. Succinic acid content was the highest in 11C, 2.06 g/L, and the lowest in 10T, 1.38g/L. The content of glacial acetic acid was 10C > 11C > 9C > 11T > 10T > 9T.

**Figure 3:**
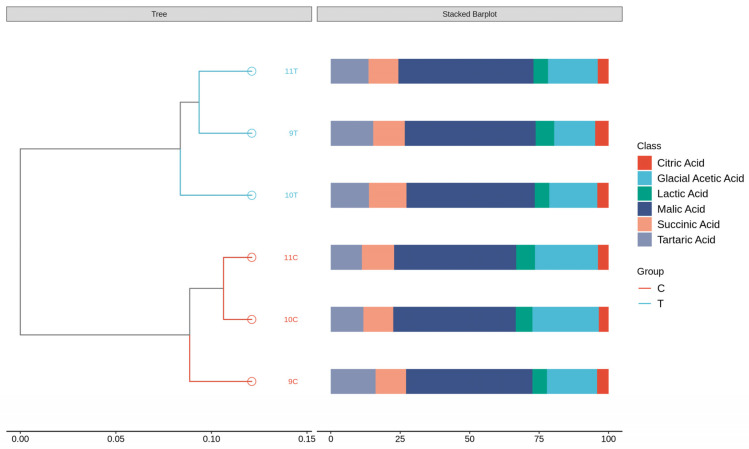
Cluster analysis of organic acids in six ice wine samples and stacked histograms of organic acid content.

As shown in Figure 3, hierarchical cluster analysis (HCA) classified the six samples into two different groups: 9C, 10C, 11C (control group) versus 9T, 10T, 11T (freeze-treated group). According to the HCA results, the characteristics of organic acid substances in different ice wine samples can be better reflected. The characteristics of the frozen wine samples and the control wine samples were different, and in combination with the organic acid content in Table 4, it can be seen that the organic acid content of the frozen wine samples was lower than that of the control wine samples. Both the composition and concentration of organic acids had an effect on the quality of the ice wine. The bar chart further shows the different proportional distribution of the six organic acids in the different samples, i.e., the compositional structures accounted for different percentages.

### 3.3. HS-GC-IMS Analysis of Beibinghong Ice Wines Under Different Treatments After Fermentation

#### 3.3.1. Qualitative Analysis of Volatile Organic Compounds (VOCs)

**Figure 4:**
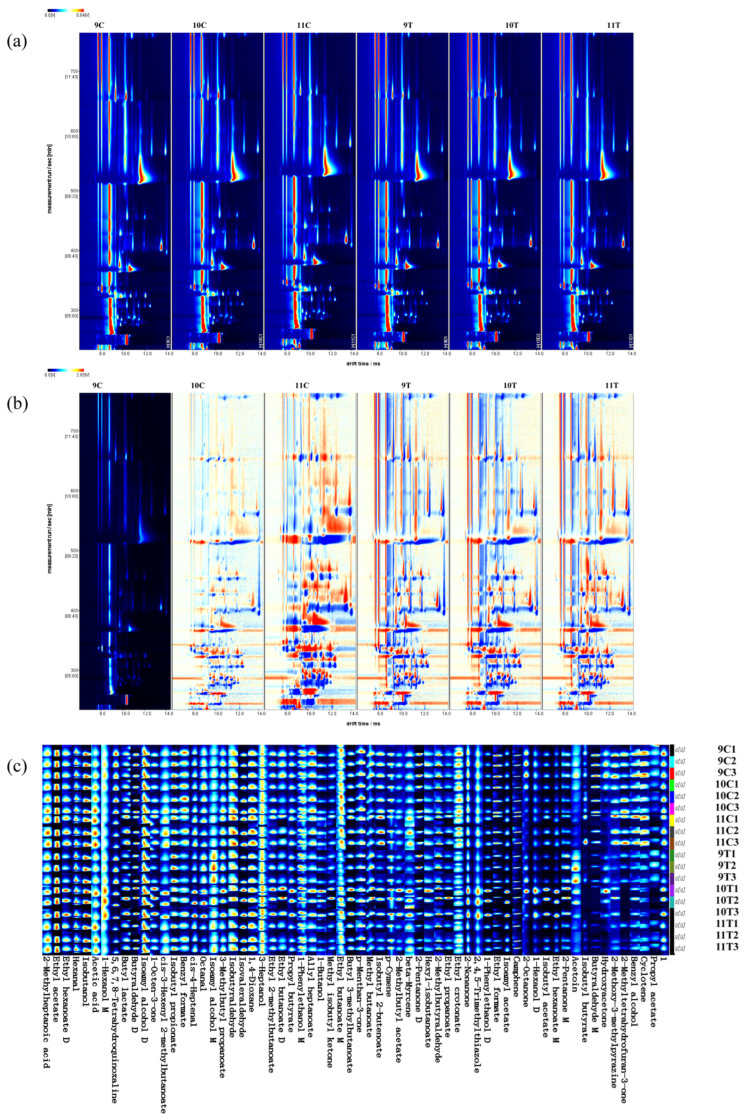
HS-GC-IMS-based qualitative analysis of 16 °C control and −20 °C treated ice wine. (**a**) Topographic map. (**b**) Differential comparison map (9C as reference). (**c**) Volatile fingerprints generated by Gallery Plot.

In order to elucidate the differences and changes in volatile compounds in different treatments after fermentation of Beibinghong ice wine, 9C was selected as the reference and other spectra were deducted from the reference to establish a GC-IMS difference chromatogram. In the deduction chromatogram, white indicates that the concentration of the substance is equal to the reference, red indicates that the concentration of the substance is higher than the reference, blue indicates that the concentration of the substance is lower than the reference, and a darker color indicates greater difference. As can be seen from Figure 4b, 11C exhibits significant divergence from other groups, whereas the differentiation of 10C is less evident compared to 9C.

The ion mobility spectra generated by the Reporter plug-in in VOCal software are shown in Figure 4a. The vertical axis represents the GC retention time and the horizontal axis corresponds to the ion migration time. Each point on either side of the reactive ion peak (RIP) represents a volatile organic compound, and the shade of the color reflects the concentration of the compound: blue indicates low content and red high content. The types of volatile compounds in the graphs are basically the same, but the colors of the points representing VOCs in the spectra are different, and the content of volatile compounds in different treatments of Beibinghong ice wine after fermentation are also different.

Two-dimensional spectra showed variations in volatile compounds, but did not allow direct identification of individual flavor components. To address this issue, we performed qualitative analysis using plug-ins to identify specific compounds and generated fingerprints. NIST database analysis confirmed 64 signal peaks, including 56 individual volatile compounds, 7 dimeric compounds, and 1 unknown substance. The color intensity reflected the concentration of the compounds. As shown in Figure 4c, there were substances with higher content in each ice wine sample, such as benzyl alcohol and propyl acetate in 9C, benzyl formate, butyl lactate, and other substances in 10C, 11C isobutanol, cis-4-heptenal, and other substances with higher content, isoamyl alcohol, 2-pentanone M, and other substances with higher content in 9T, and 3-heptanol, isovaleraldehyde, and other substances with higher content in 10T.

#### 3.3.2. Volatile Component Profiling

Qualitative analysis via the Library Search plugin in VOCAL identified 56 volatile compounds across all six ice wine samples, with consistent compound categories observed. The compositional breakdown was as follows: 23 esters, 10 ketones, 7 alcohols, 7 aldehydes, 2 terpenes, 2 acids, and 5 miscellaneous compounds.

Peak volume integration of volatile organic compounds was performed using the VOCal plug-in, and relative quantification was performed by the internal standard method (see Section 2.2.5 for details of the calculation methodology), in which 10T had the highest concentration of volatile compounds with 32,053.06 µg/L, followed by 9C with 31,636.64 µg/L, and the lowest being 11T with 28,965.38 µg/L. The percentage of each volatile compound varied among the samples, with alcohols accounting for the largest percentage, ranging from 37.28% to 40.18%. The specific volatile compound concentrations of the six ice wine samples are shown in Table 5.

As shown in Table 5, alcohols are the most impactful aromatic compounds in wine, contributing both ethanol-derived notes and distinctive aromas. For instance, isoamyl alcohol imparts a whiskey-like nuance, while benzyl alcohol exhibits cherry-like fragrance [32]. Among the detected alcohols in the ice wine samples, 9C contained 11,794.37 µg/L, 10C contained 12,073.03 µg/L, 11C contained 11,976.22 µg/L, 9T contained 11,648.03 µg/L, 10T contained 12,537.05 µg/L,11T contained 11,518.61 µg/L, 10T had the highest content, and 9T had the lowest content.

Esters are mainly produced during wine fermentation in the presence of yeasts and bacteria, with small amounts originating from aging [33]. Esters are also a key factor in the aroma of wine, which usually has a pleasant odor [34]. As shown in Table 6, among the esters detected in the ice wine samples, 10T had the highest content of 9813.35 µg/L, followed by 11C with 9650.61 µg/L, 9C with 9348.80 µg/L, 10C with 9267.49 µg/L, 11T with 8074.71 µg/L, and 9T with 8074.71 µg/L.

Aldehydes are usually associated with the oxidation process during fermentation and can bring out a peculiar odor [35]. The aldehyde content was ranked as 11C > 10C > 9C > 10T > 11T > 9T, with the highest value being 11C at 4937.21 µg/L.

The total concentrations of ketones, acids, terpenes, and other substances were small, accounting for only 5.72%–8.59%, 3.25%–4.23%, 0.51%–0.6%, and 3.93%–5.71% of each ice wine sample.

**Table 5:** Composition of volatile compounds in Beibinghong ice wine.

	CAS	Substance Name	Volatile Compound Content (µg·L^−1^)					
			9C	10C	11C	9T	10T	11T
1	111-27-3	1-Hexanol D	199.88 ± 23.69b	171.73 ± 21.54bc	159.37 ± 9.63bcd	122.25 ± 9.2cd	283.23 ± 63.42a	105.95 ± 11.16d
2	111-27-3	1-Hexanol M	125.76 ± 10.03bc	119.05 ± 4.57cd	114.45 ± 2.74cd	132.98 ± 5.43b	153.06 ± 7.23a	112.69 ± 4.98d
3	78-83-1	Isobutanol	1297.35 ± 69.74ab	1243.34 ± 58.25b	1385.81 ± 26.73a	1331.54 ± 9.03ab	1326.58 ± 41.33ab	1255.03 ± 57.98b
4	123-51-3	Isoamyl alcohol D	5799.69 ± 94.64ab	5896.59 ± 36.44a	5660.01 ± 119.94bc	5577.88 ± 54.54c	5664.45 ± 117.99bc	5544.99 ± 123.97c
5	123-51-3	Isoamyl alcohol M	222.26 ± 20.25b	204.52 ± 20.82b	214.02 ± 17.2b	311.53 ± 13.47a	243.08 ± 38.52b	234.24 ± 17.3b
6	589-82-2	3-Heptanol	3115.8 ± 181.88c	3385.68 ± 58.87b	3344.27 ± 28.01bc	3192.78 ± 79.22bc	3670.47 ± 80.65a	3208.59 ± 240.56bc
7	100-51-6	Benzyl alcohol	29.29 ± 2.2a	23.39 ± 3.29b	27.07 ± 2.5ab	17.86 ± 1.25c	17.47 ± 1.36c	19.44 ± 1.34c
8	71-36-3	1-Butanol	109.94 ± 20.44b	106.7 ± 14.09b	142.19 ± 6.8a	97.95 ± 0.95b	148.51 ± 15.82a	102.95 ± 15.82b
9	98-85-1	1-Phenylethanol D	228.61 ± 7.65e	245.82 ± 9.74d	293.02 ± 8.04b	204.76 ± 1.86f	393.35 ± 9.49a	270.6 ± 7.28c
10	98-85-1	1-Phenylethanol M	665.78 ± 10.87a	676.19 ± 3.16a	636.02 ± 73.95a	658.5 ± 11.31a	636.86 ± 44.76a	664.14 ± 14.03a
**Alcohols**	**7**	**Subtotal**	**11,794.36521**	**12,073.03437**	**11,976.218**	**11,648.025**	**12,537.052**	**11,518.613**
		**Percentage**	**37.28%**	**38.60%**	**38.17%**	**40.18%**	**39.11%**	**39.77%**
1	6728-31-0	cis-4-Heptenal	754.17 ± 44.95b	744.46 ± 47.94b	829.53 ± 47.84a	658.53 ± 18.13c	742.13 ± 33.25b	747.98 ± 21.64b
2	66-25-1	Hexanal	1507.6 ± 46.12abc	1527.8 ± 32.27abc	1559.4 ± 36.74a	1540.04 ± 21ab	1465.38 ± 33.31c	1485.65 ± 32.5bc
3	96-17-3	2-Methylbutyraldehyde	360.16 ± 19.24a	340.72 ± 15.81ab	320.57 ± 29ab	323.65 ± 6.78ab	306.26 ± 31.56b	308.82 ± 17.4b
4	78-84-2	Isobutyraldehyde	524.14 ± 6.31a	504.32 ± 49.78ab	538.94 ± 15.1a	454.52 ± 29.71bc	427.14 ± 27.18c	443.01 ± 47.91bc
5	123-72-8	Butyraldehyde D	547.21 ± 10.8a	501.38 ± 9.04bc	494.28 ± 35.25c	479.52 ± 11.73cd	531.27 ± 14.48ab	459.73 ± 14.48d
6	123-72-8	Butyraldehyde M	25.57 ± 0.63a	23.43 ± 0.86ab	20.25 ± 4.12b	12.7 ± 0.43c	7.35 ± 3.46d	10.22 ± 0.2cd
7	590-86-3	Isovaleraldehyde	601.82 ± 48.78d	796.46 ± 21.21c	867.62 ± 13.95ab	573.12 ± 3.54d	888.13 ± 34.7a	819.94 ± 9.29bc
8	124-13-0	Octanal	453.16 ± 25.93a	396.56 ± 60.14ab	306.63 ± 27.68c	408.57 ± 11.98ab	357.09 ± 24.09bc	234.65 ± 22.87d
**Aldehydes**	**7**	**Subtotal**	**4773.840547**	**4835.133622**	**4937.2073**	**4450.6514**	**4724.7541**	**4509.9855**
		**Percentage**	**15.09%**	**15.46%**	**15.73%**	**15.35%**	**14.74%**	**15.57%**
1	80-71-7	Cyclotene	37.92 ± 2.86a	28.41 ± 3.4b	38.7 ± 0.97a	23.67 ± 0.72b	18.17 ± 2.33c	23.81 ± 3.97b
2	107-87-9	2-Pentanone D	77.72 ± 4.46a	69.01 ± 1.53b	79.03 ± 5.07a	79 ± 5.65a	64.5 ± 2.35b	53.47 ± 6.8c
3	107-87-9	2-Pentanone M	109.39 ± 3.78a	75.51 ± 2.87b	76.9 ± 8.15b	115.01 ± 5.22a	112.21 ± 11.14a	80.01 ± 4.77b
4	821-55-6	2-Nonanone	591.72 ± 33.09a	568.17 ± 21.24ab	523.61 ± 19.33b	432.23 ± 15.04c	607.75 ± 61.2a	433.09 ± 13.78c
5	3188-00-9	2-Methyltetrahydrofuran-3-one	37.3 ± 8.31a	44.22 ± 9.32a	38.83 ± 10.82a	22.5 ± 2.11b	4.59 ± 0.68c	14.05 ± 6.19bc
6	111-13-7	2-Octanone	1083.92 ± 91.43a	703.68 ± 167.84bc	527.45 ± 17.58cd	762.85 ± 111.42b	818.4 ± 188.83b	454.4 ± 20.06d
7	513-86-0	Acetoin	59.38 ± 8.06ab	38.89 ± 13.63cd	33.34 ± 4.1cd	73.71 ± 10.6a	45.19 ± 8.27bc	26.66 ± 3.65d
8	108-10-1	Methyl isobutyl ketone	113.24 ± 10.93a	84.08 ± 11.26b	117.44 ± 16.4a	115.74 ± 15.49a	102.52 ± 10.58ab	98.55 ± 15.21ab
9	4312-99-6	1-Octen-3-one	116.21 ± 7.28b	124.75 ± 2.68b	166.3 ± 16.14a	88.61 ± 6.32c	172 ± 19.77a	118.29 ± 7.21b
10	116-09-6	Hydroxyacetone	145.65 ± 25.55a	82.29 ± 28.07bc	47.74 ± 7.58c	117.03 ± 23.15ab	108.96 ± 36.68ab	47.03 ± 4.49c
11	10458-14-7	p-Menthan-3-one	345.02 ± 20.26a	337.44 ± 12.12ab	314.76 ± 12.52ab	272.23 ± 13.95c	299.24 ± 7.75bc	307.56 ± 38.38abc
**Ketones**	**10**	**Subtotal**	**2717.459965**	**2156.455909**	**1964.0937**	**2102.5693**	**2353.5328**	**1656.9252**
		**Percentage**	**8.59%**	**6.89%**	**6.26%**	**7.25%**	**7.34%**	**5.72%**
1	142-19-8	Allyl heptanoate	80.74 ± 2.05a	76.78 ± 0.88ab	69.64 ± 5.43b	59.61 ± 6.48c	57.55 ± 8.14c	56.87 ± 4.4c
2	104-57-4	Benzyl formate	64.7 ± 7.39ab	69.21 ± 5.92a	61.76 ± 4.7ab	45.42 ± 2.16c	60.42 ± 1.44ab	56.39 ± 5.75b
3	105-66-8	Propyl butyrate	401.38 ± 29.61bc	392.62 ± 24.8bc	402.83 ± 2.48bc	414.24 ± 5.39b	456.35 ± 34.72a	360.76 ± 16.21c
4	109-19-3	Butyl 3-methylbutanoate	185.33 ± 5.31b	181.24 ± 9.08b	213.11 ± 2.14a	168.51 ± 2.84b	227.71 ± 18.19a	176.32 ± 9.81b
5	138-22-7	Butyl lactate	198.73 ± 12.07ab	216.09 ± 4.83a	184.25 ± 22.5bc	156.26 ± 8.54c	170.28 ± 15.14c	177.62 ± 18.22bc
6	53398-85-9	cis-3-Hexenyl 2-methylbutanoate	205.29 ± 7.55ab	216.85 ± 12.88a	219.62 ± 23a	168.12 ± 11.71c	182.57 ± 17.22bc	213.09 ± 21.54ab
7	7452-79-1	Ethyl 2-methylbutanoate	207.4 ± 5.94a	192.5 ± 5.68a	180.73 ± 24.32a	193.6 ± 5.2a	204.37 ± 21.56a	186.9 ± 4.2a
8	141-78-6	Ethyl acetate	3208.39 ± 4.87c	3258.77 ± 21.19b	3311.42 ± 5.27a	2979.23 ± 24.46e	3292.77 ± 30.1a	3149.88 ± 7.07d
9	105-54-4	Ethyl butanoate D	63.02 ± 6.92a	43.41 ± 7.16b	50.54 ± 7.46b	47.34 ± 3.91b	49.6 ± 5.33b	44.79 ± 5.22b
10	105-54-4	Ethyl butanoate M	589.76 ± 33.32b	675.47 ± 50.29a	596.29 ± 43.81b	448.82 ± 4.34c	572.29 ± 24.5b	553.95 ± 59.08b
11	624-41-9	2-Methylbutyl acetate	101.37 ± 31.17ab	104.94 ± 20.65ab	113.65 ± 7.58ab	113.73 ± 4.18ab	129.89 ± 12.17a	87.6 ± 12.14b
12	105-68-0	3-Methylbutyl propanoate	455.99 ± 40.74a	443.07 ± 41.51ab	445.4 ± 33.85ab	437.42 ± 6.11abc	374.34 ± 47.96c	388.32 ± 14.85bc
13	623-70-1	Ethyl crotonate	221.17 ± 4.63b	225.54 ± 4.95b	244.81 ± 18.87a	173.23 ± 5.38d	185.69 ± 10.06cd	198.26 ± 6.7c
14	109-94-4	Ethyl formate	69.32 ± 2.66a	69.38 ± 3.17a	57.88 ± 5.05b	53.78 ± 2.92b	35.89 ± 7.21c	43.36 ± 6.82c
15	123-66-0	Ethyl hexanoate M	195.1 ± 12.82ab	200.09 ± 14.21ab	209.37 ± 14.9a	134.73 ± 7.42c	188.17 ± 6.52ab	178.93 ± 13.29b
16	123-66-0	Ethyl hexanoate D	610.99 ± 40.19c	562.74 ± 64.65c	720.16 ± 92.36b	369.94 ± 11.59d	1005.18 ± 89.01a	590.01 ± 4.37c
17	105-37-3	Ethyl propanoate	55.24 ± 8.55a	54.81 ± 5.61a	53.55 ± 7.32ab	44.01 ± 2.89b	50.87 ± 3.63ab	46.08 ± 1.34ab
18	123-92-2	Isoamyl acetate	1027.89 ± 19.33d	1084.86 ± 44.3d	1246.27 ± 49.87b	914.26 ± 15.77e	1429.63 ± 69.91a	1162.46 ± 16.69c
19	589-66-2	Isobutyl 2-butenoate	57.97 ± 9.05b	59.55 ± 3.34b	72.8 ± 5.18a	40.36 ± 0.68c	49.59 ± 2.08bc	44.04 ± 6.92c
20	110-19-0	Isobutyl acetate	122.84 ± 5.82c	97.85 ± 6.57d	134.26 ± 2.48b	76.14 ± 4.08e	174.42 ± 9.83a	106.31 ± 5.36d
21	539-90-2	Isobutyl butyrate	219.47 ± 63.72a	186.24 ± 74.98ab	183.32 ± 110.02ab	100.96 ± 52.19ab	71.13 ± 8.99b	113.68 ± 32.42ab
22	540-42-1	Isobutyl propionate	620.55 ± 17.88a	570.82 ± 24bc	595.17 ± 16.77ab	591.66 ± 6.81ab	558.12 ± 31.3bc	538.16 ± 16.17c
23	623-42-7	Methyl butanoate	280.59 ± 14.91a	202.8 ± 15.07cd	207.24 ± 1.75cd	246.96 ± 14.1b	220.16 ± 16.08c	194.94 ± 7.83d
24	2349/7/7	Hexyl isobutanoate	51.68 ± 6.63a	49.29 ± 6.18ab	54.4 ± 3.61a	48.44 ± 2.63ab	56.1 ± 7.31a	40.28 ± 4.26b
25	109-60-4	Propyl acetate	53.9 ± 10.45a	32.55 ± 6.48b	22.12 ± 9.7bc	47.95 ± 3.41a	10.26 ± 1.75c	31.85 ± 3.99b
**Esters**	**23**	**Subtotal**	**9348.804446**	**9267.492554**	**9650.6059**	**8074.71**	**9813.3474**	**8740.8389**
		**Percentage**	**29.55%**	**29.63%**	**30.76%**	**27.85%**	**30.62%**	**30.18%**
1	123-35-3	beta-Myrcene	51.91 ± 1.26b	51.88 ± 6.73b	74.27 ± 5.62a	38.68 ± 2.91c	75.7 ± 7.31a	62.17 ± 6.98b
2	79-92-5	Camphene	116.02 ± 5.38a	112.3 ± 3.42a	114.31 ± 27.03a	108.41 ± 4.04a	94.2 ± 14a	112.48 ± 3.89a
**Terpenoids**	**2**	**Subtotal**	**167.9257814**	**164.1848294**	**188.58409**	**147.08833**	**169.89666**	**174.64383**
		**Percentage**	**0.53%**	**0.52%**	**0.60%**	**0.51%**	**0.53%**	**0.60%**
1	97-61-0	2-Methylvaleric acid	464.77 ± 12.56b	497 ± 26.83b	582.88 ± 53.99a	485.18 ± 6.31b	462.73 ± 21b	516.32 ± 35.27b
2	64-19-7	Acetic acid	561.98 ± 53.52c	686.54 ± 41.26ab	726.32 ± 19.7a	591.31 ± 9.19c	627.88 ± 93.92bc	708.54 ± 17.28ab
**Acids**	**2**	**Subtotal**	**1026.748502**	**1183.531943**	**1309.1985**	**1076.4904**	**1090.6044**	**1224.8565**
		**Percentage**	**3.25%**	**3.78%**	**4.17%**	**3.71%**	**3.40%**	**4.23%**
1	2847-30-5	2-Methoxy-3-methylpyrazine	16.5 ± 3.18a	12.37 ± 1.14bc	14.8 ± 1.86ab	9.39 ± 1.8cd	9.68 ± 0.46cd	8.38 ± 1.37d
2	99-87-6	p-Cymene	230.51 ± 11.33d	255.05 ± 6.03cd	299.78 ± 9.88ab	272.3 ± 0.64bc	332.34 ± 48.77a	288.92 ± 9.57bc
3	123-91-1	1,4-Dioxane	167.83 ± 1.06b	177.51 ± 7.17b	198.93 ± 2.74a	151.81 ± 4.27c	172.14 ± 4.61b	178.71 ± 11.08b
4	13623-11-5	2,4,5-Trimethylthiazole	132.26 ± 12.6b	124.59 ± 6.92b	132.71 ± 0.4b	94.35 ± 3.33c	175.72 ± 8.3a	102.03 ± 2.57c
5	34413-35-9	5,6,7,8-Tetrahydroquinoxaline	1260.39 ± 44.09a	1029.45 ± 99.62b	705.92 ± 37.1c	961.39 ± 83.91b	673.99 ± 95.66cd	561.47 ± 34.96d
**Others**	**5**	**Subtotal**	**1807.49296**	**1598.96815**	**1352.1458**	**1489.2449**	**1363.8739**	**1139.5156**
		**Percentage**	**5.71%**	**5.11%**	**4.31%**	**5.14%**	**4.26%**	**3.93%**
		**Total**	**31,636.63742**	**31,278.80138**	**31,378.053**	**28,988.78**	**32,053.062**	**28,965.379**

Different lowercase letters indicate significant differences between treatments (Duncan’s test *p* < 0.05). _M and _D are the monomer and dimer of the same substance.

**Table 6:** OAV analysis of volatile compounds in different ice wine samples. Volatile compounds OAV > 1.

Volatile Flavor Compounds	Aroma Profile ^a^	Odor Threshold (mg/kg)	Literature	OAV					
				9C	10C	11C	9T	10T	11T
1-Hexanol	Banana, Flower, Grass, Herb	0.0056	b	58.15	51.93	48.90	45.58	77.91	39.04
Isobutanol	Apple, Bitter, Cocoa, Wine	0.55	b	2.36	2.26	2.52	2.42	2.41	2.28
Isoamyl alcohol	Burnt, Cocoa, Floral, Malt	0.004	b	1505.49	1525.28	1468.51	1472.35	1476.88	1444.81
3-Heptanol	Herb	0.24	b	12.98	14.11	13.93	13.30	15.29	13.37
1-Phenylethanol	Floral, Honey, Rose	0.56423	b	1.59	1.63	1.65	1.53	1.83	1.66
cis-4-Heptenal	Dairy	0.00006	b	12,569.43	12,407.60	13,825.53	10,975.48	12,368.79	12,466.36
Hexanal	Apple, Fat, Fresh, Green, Oil	0.005	b	301.52	305.56	311.88	308.01	293.08	297.13
2-Methylbutyraldehyde	Almond, Cocoa, Fermented, Hazelnut, Malt	0.001	b	360.16	340.72	320.57	323.65	306.26	308.82
Isobutyraldehyde	Burnt, Caramel, Cocoa, Green, Malt	0.0015	b	349.43	336.21	359.29	303.01	284.76	295.34
Butyraldehyde	Banana, Green, Pungent	0.002	b	286.39	262.41	257.26	246.11	269.31	234.97
Isovaleraldehyde	Ethereal, Cocoa, pineapple	0.0046	b	130.83	173.14	188.61	124.59	193.07	178.25
Octanal	Citrus, Fat, Green, Oil, Pungent	0.000587	b	772.00	675.57	522.37	696.03	608.34	399.74
2-Nonanone	Fragrant, Fruit, Green, Hot Milk	0.041	b	14.43	13.86	12.77	10.54	14.82	10.56
2-Methyltetrahydrofuran-3-one	Nuts	0.04	[36]	<1	1.11	<1	<1	<1	<1
2-Octanone	Fat, Fragrant, Mold	0.0502	b	21.59	14.02	10.51	15.20	16.30	9.05
Acetoin	Butter, Creamy, Green Pepper	0.014	b	4.24	2.78	2.38	5.26	3.23	1.90
1-Octen-3-one	Earth, Mushroom	0.004	b	29.05	31.19	41.58	22.15	43.00	29.57
p-Menthan-3-one	Green, Fresh, Mint	0.21	b	1.64	1.61	1.50	1.30	1.42	1.46
Propyl butyrate	Apricot, Fruit, Pineapple, Solvent	0.018	b	22.30	21.81	22.38	23.01	25.35	20.04
Ethyl 2-methylbutanoate	Apple, Ester, Green Apple, Kiwi, Strawberry	0.000013	b	15,953.69	14,807.96	13,902.18	14,892.25	15,720.85	14,377.28
Ethyl acetate	Aromatic, Brandy, Grape	0.005	b	641.68	651.75	662.28	595.85	658.55	629.98
Ethyl butanoate	Apple, Butter, Cheese, Pineapple, Strawberry	0.0009	b	725.31	798.76	718.69	551.29	690.99	665.26
2-Methylbutyl acetate	Apple, Banana, Pear	0.005	b	20.27	20.99	22.73	22.75	25.98	17.52
3-Methylbutyl propanoate	Apple, Apricot, Pineapple	0.0086	b	53.02	51.52	51.79	50.86	43.53	45.15
Ethyl crotonate	Tropical Fruit	0.0025	b	88.47	90.22	97.92	69.29	74.28	79.30
Ethyl hexanoate	Apple Peel, Brandy, Fruit Gum, Overripe Fruit, Pineapple	0.0022	b	366.40	346.74	422.51	229.40	542.43	349.52
Ethyl propanoate	Apple, Pineapple, Rum, Strawberry	0.01	b	5.52	5.48	5.36	4.40	5.09	4.61
Isoamyl acetate	Apple, Banana, Pear	0.019	b	54.10	57.10	65.59	48.12	75.24	61.18
Isobutyl acetate	Apple, Banana, Floral, Herb	0.025	b	4.91	3.91	5.37	3.05	6.98	4.25
Methyl butanoate	Apple, Banana, Cheese, Ester, Floral	0.059	b	4.76	3.44	3.51	4.19	3.73	3.30
Hexyl isobutanoate	Fruit	0.00007	[37]	738.22	704.15	777.13	691.99	801.40	575.36
beta-Myrcene	Balsamic, Fruit, Geranium, Herb, Must	0.0012	b	43.26	43.24	61.89	32.23	63.08	51.81
2-Methoxy-3-methylpyrazine	Nuts	0.007	b	2.36	1.77	2.11	1.34	1.38	1.20
p-Cymene	Citrus, Fresh, Solvent	0.00501	b	46.01	50.91	59.84	54.35	66.34	57.67

Notes: a: Aroma descriptions from the website: https://www.femaflavor.org/; https://www.chemicalbook.com/ (accessed on 3 November 2024); b: Aroma thresholds from the book: “ODOUR THRESHOLDS”.

### 3.4. Differential Analysis of Volatile Profiles in Post-Fermentation Treated Beibinghong Ice Wines

**Figure 5:**
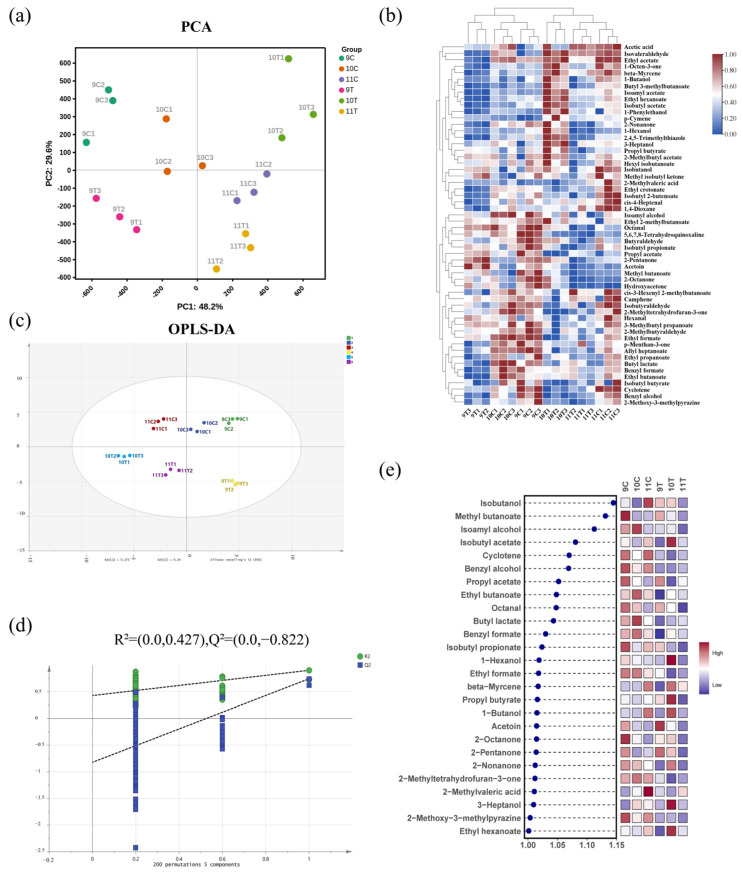
Analysis of volatile compound content in six ice wine samples. (**a**) PCA plot of volatile compound content; (**b**) hierarchical clustering heatmap of volatile compounds (note: color gradient from blue to red indicates increasing concentration of volatile compounds, redder hues denote higher content, bluer hues denote lower content); (**c**) OPLS-DA score plot of volatile compound content; (**d**) cross-validation results from 200 permutation tests; (**e**) VIP plot from OPLS-DA analysis based on volatile compound content (*p* < 0.05).

The results of principal component analysis (PCA) based on the characteristics of volatile compound content in Figure 5a showed that the cumulative explanatory rate of the first two principal components reached 77.8%, of which the variance contributions of PC1 and PC2 were 48.2% and 29.6%, respectively. There was no large overlap in the area of distribution of the samples, which showed a significant trend of separation and good clustering within each sample group. This indicates that the volatile compound content of the six samples is significantly different.

Clustering heatmap analysis effectively revealed the distribution patterns of volatile compounds and inter-sample relationships (Figure 5b). The 9% alcohol samples (9T, 9C) clustered with 10C, 10T grouped with the 11% alcohol samples (11T, 11C), and 10T did not cluster with 10C, indicating distinct volatile compound profiles in 10% alcohol samples subjected to freezing treatment compared to controls.

The OPLS-DA model, using 56 shared volatile compounds as dependent variables and 6 ice wine samples as independent variables, demonstrated robust performance (R^2^X = 0.906, R^2^Y = 0.891, Q^2^ = 0.742; Figure 5c). A 200-permutation test validated the model’s effectiveness, with the Q^2^ regression line intersecting below zero on the vertical axis (Figure 5d), confirming no overfitting. This model reliably differentiated ice wine samples based on volatile profiles.

Screening with VIP > 1 and *p* < 0.05 identified 27 key differential metabolites (Figure 5e). Among the key difference secondary metabolites, there were ten esters, sic alcohols, six ketones, one aldehyde, one acid, one terpene, and one other. The only key differential secondary metabolite among the seven aldehydes detected was octanal, indicating that most of the aldehydes were similar between the freezing treatment and the control treatment. The freezing treatment did not alter the content of most of the aldehydes.

### 3.5. Differential Flavor Profiles of Post-Fermentation Treated Beibinghong Ice Wines

The content of volatile compounds affects the overall aroma of a wine, but does not directly reflect the effect of volatile compounds on the overall aroma of a sample. The overall aroma of wine is related to the perception threshold of each compound, which varies greatly among different volatile compounds. OAV is the ratio of the concentration of an aroma component to the odor threshold, and in recent years the use of OAV has allowed the identification of many odors in wine [38], so by calculating the odor activity value of each volatile compound, the flavor profiles of different ice wine samples were derived, as well as the differences of volatile compounds that affect the differences in ice wine samples of the key volatile compounds.

Notes: Aroma descriptions from the website: https://www.femaflavor.org/; https://www.chemicalbook.com/ (accessed on 3 November 2024): Aroma thresholds from the book: “ODOUR THRESHOLDS”. A total of 34 volatile flavor substances with OAV > 1 were detected in the six ice wine samples, with a total of five alcohols, seven aldehydes, six ketones, thirteen esters, one terpene, and two others (Table 6). Among them, 2-methyltetrahydrofuran-3-one was greater than 1 in 10C and less than 1 in the rest of the wine samples. The ten constituents with the highest OAV values were five esters, one alcohol, and four aldehydes, namely ethyl 2-methylbutanoate, cis-4-heptenal, and isoamyl. The specific organoleptic properties of the esters were as follows: among the esters, ethyl 2-methylbutanoate, the core flavor substance, significantly enhanced the fruity quality by balancing sweetness and sensory comfort [39]. Hexyl isobutanoate, ethyl butanoate, and ethyl acetate synergistically constructed a multilayered fruity flavor profile [40,41]. Among the aldehyde fractions, cis-4-heptenal (fatty green aroma) suggests a possible oxidative metabolism pathway [42], octanal (fruity-honey aroma) and 2-methylbutyraldehyde (sweet aroma) work together to intensify the sweetness hierarchy [43], and the malty-green aroma of isobutyraldehyde creates an olfactory contrast [44]. Isoamyl alcohol is the key alcohol, and its characteristic whiskey aroma gives a rich tone to the spirit [32]. The above compounds build the main distinctive aroma profile characteristics of ice wine through the synergistic effect of the three sensory attributes of sweetness, fruity, and green aromas.

**Figure 6:**
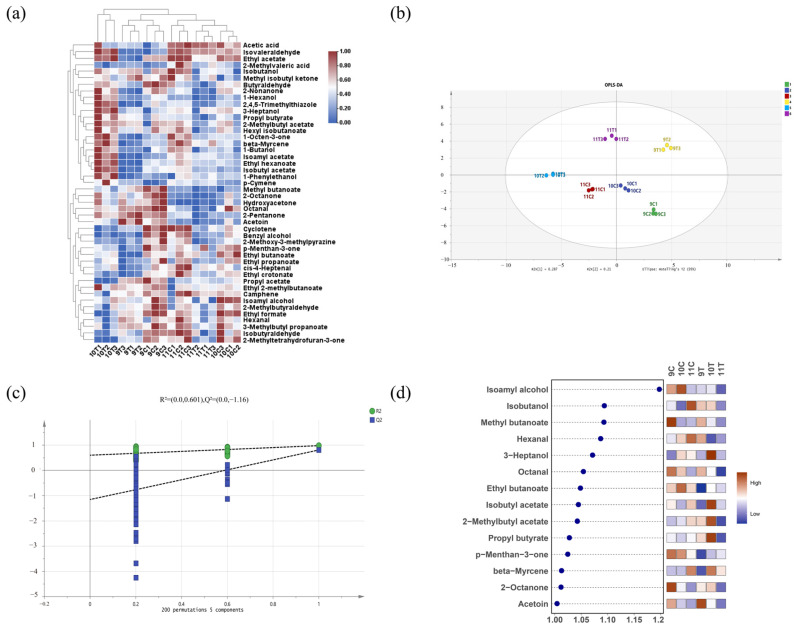
Analysis of odor activity value (OAV) for volatile compounds in six ice wine samples. (**a**) Hierarchical clustering heatmap of volatile compound OAV (note: color gradient from blue to red indicates increasing OAV; redder hues denote higher OAV, bluer hues denote lower OAV); (**b**) OPLS-DA score plot of volatile compound OAV; (**c**) cross-validation results from 200 permutation tests; (**d**) VIP plot from OPLS-DA analysis based on volatile compounds with OAV > 1 (*p* < 0.05).

In order to further verify the differences in aroma composition of different treated ice wine samples after fermentation, a clustering heatmap was drawn for the 34 volatile flavor substances with OAV > 1. The 34 volatile flavor substances with OAV > 1 could be classified into three groups for the six samples: 10T was clustered independently, which was characteristically enriched with 16 aroma compounds such as 2-nonanone and 1-hexanol; 9C and 9T formed a secondary cluster with methyl butanoate, 2-octanone, and other markers; 10C, 11C, and 11T were clustered into the third category through common components such as acetic acid (Figure 6a). From these grouping results, it can be seen that except for 10T, the aroma compounds in the freezing-treated group (9T, 11T) and the control group (9C, 10C, 11C) showed cross-clustering characteristics, indicating that the freezing process did not lead to a significant loss in flavor components.

To identify key differential aroma compounds in post-fermentation ice wines, OPLS-DA analysis (R^2^X = 0.95, R^2^Y = 0.957, Q^2^ = 0.795) was conducted using volatiles with OAV > 1, and the model passed permutation tests (200 cycles; Figure 6c). The intersection of the Q^2^ regression line with the vertical axis below zero confirmed no overfitting, validating the model. Figure 6d details the VIP scores, identifying fourteen key differential aroma compounds (VIP > 1, *p* < 0.05), including five esters, three ketones, three alcohols, two aldehydes, and one terpene, which are critical for differentiating ice wine aromas. The top-five VIP-ranked compounds were isoamyl alcohol, isobutanol, methyl butanoate, hexanal, and 3-heptanol. Among the top-five VIP values, methyl butanoate of 9C was the most prominent key differential aroma component, which is mainly fruity and floral according to the aroma description on the online website. Isoamyl alcohol of 10C was the most prominent key differential aroma component, which is mainly cocoa and floral. Isobutanol and hexanal of 11C were the most prominent key differential aroma components, which are mainly cocoa, fruity and wine. 3-heptanol of 10T was the most prominent key differential aroma component, which is mainly herbaceous. Isobutanol and hexanal of 11C were the most prominent, mainly presenting cocoa, fruit, and wine aroma. 3-heptanol of 10T is the most prominent key differential aroma component, mainly presenting herbaceous aromas.

### 3.6. Sensory Evaluation of Beibinghong Ice Wines with Different Treatments After Fermentation

In this study, sensory evaluation and flavor analysis of ice wine samples with different treatments after fermentation were carried out. The sensory scores showed (Table 7) that all the samples performed well in terms of color and clarity (score ≥ 9.0), with the 10T group leading the way with a composite score of 91.29, showing the best performance in terms of delicate aroma, rich layering, balanced taste, and typicality.

**Figure 7:**
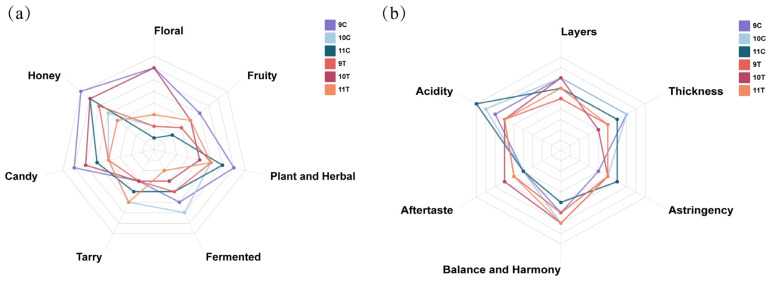
Sensory profiling of six ice wine samples. (**a**) Aroma radar chart of ice wine samples; (**b**) taste radar chart of ice wine samples.

By calculating the OAV of each volatile compound, it was determined that most aroma profiles of ice wine samples were composed of fruity, sweet, and grassy notes. The sensory attributes floral, honey, candy, fruity, plant and herbal, fermented, and tarry were used to characterize ice wine aromas. Statistical analysis of scoring results (Figure 7a) revealed differences in aroma intensity across samples for each descriptor: 9C exhibited prominent candy, honey, and plant and herbal aromas; 10C showed a dominant fermented aroma; 11C emphasized plant and herbal and honey aromas; 9T highlighted honey; 10T featured floral and honey; and 11T displayed prominent plant and herbal and tarry attributes. Radar chart analysis demonstrated that post-fermentation freezing treatments affected aroma intensity profiles of the samples.

Statistical analysis results of taste evaluation indicators (Figure 7b) showed that samples 11C, 10C, and 9C exhibited prominent acidity scores; 10C and 10T displayed dominant layer scores; 9T, 10T, and 11T highlighted aftertaste scores; 10C, 10T, and 11T emphasized balance and harmony scores; 11C showed prominent astringency scores; and 9C, 10C, and 11C featured notable thickness scores. Correlation analysis between taste attributes and physicochemical indicators (Supplementary Figure S1) retained indicators with absolute values > 0.8 for clarity. Significant correlations were observed: Acidity was positively correlated with total acidity, anthocyanin, tannins, and total phenols, indicating these as key parameters for evaluating ice wine acidity. Thickness was positively correlated with total sugar, total acidity, anthocyanin, tannins, and total phenols, confirming these as critical indicators for assessing ice wine thickness.

Based on the analysis of taste evaluation indicators, the three control group samples (9C, 10C, and 11C) exhibited higher acidity and thickness. In contrast, the three freeze-treated samples (9T, 10T, and 11T) showed lower acidity and thickness compared to the control group. Given the significant correlations between acidity/thickness and physicochemical parameters, it can be inferred that the freezing treatment caused a reduction in critical physicochemical components such as total acidity, tannins, anthocyanins, total phenols, and total sugar, thereby contributing to the lower acidity and thickness values observed in the freeze-treated samples. It can be seen that the post-fermentation freezing treatment improved the acidity and heaviness of the ice wine.

## 4. Conclusions

This study investigated the effects of post-fermentation freezing treatment on ice wine by analyzing basic physicochemical parameters, organic acids, volatile components, and sensory quality. The results demonstrated significant differences in physicochemical indicators between freeze-treated and control ice wine samples. Specifically, the freeze-treated samples (9T, 10T, 11T) exhibited lower levels of total sugar, tannins, and total acidity compared to the control group (9C, 10C, 11C), which alleviated overly sweet, pungent, and bitter tastes, resulting in a more harmonious and balanced flavor profile. Additionally, all six organic acid concentrations in the freeze-treated samples were reduced relative to the controls, and hierarchical cluster analysis (HCA) confirmed distinct clustering patterns between the two groups.

The volatile components in post-fermentation treated Beibinghong ice wine were analyzed using headspace gas chromatography–ion mobility spectrometry (HS-GC-IMS). A total of 56 volatile compounds were identified: 23 esters, 7 alcohols, 7 aldehydes, 10 ketones, 2 terpenes, 2 acids, and 5 other compounds. Principal component analysis (PCA) and clustering heatmaps indicated differences in volatile compound profiles among the six samples. Orthogonal partial least squares discriminant analysis (OPLS-DA) and screening of key differential flavor compounds (VIP > 1, *p* < 0.05) revealed that freezing treatment altered the content of specific esters, alcohols, and ketones. Calculation of odor activity values (OAV) identified 34 volatile compounds with OAV > 1, and the top 10 aroma descriptors (sweet, fruity, and herbal notes) confirmed that freezing preserved the core aroma profile. OPLS-DA and VIP values further identified 14 key aroma compounds distinguishing ice wine samples, with descriptors including floral, fruity, herbal, and wine-like notes.

From the results of sensory scores, 10T had the highest score, followed by 9C, 9T, 11T, 11C, and 10C. According to the results of aroma evaluation, there were differences in the aroma intensity of each descriptor in the samples, suggesting that the post-fermentation freezing treatment affected the aroma intensity of the samples. Based on sensory evaluation and correlation analysis, the three freezing-treated samples (9T, 10T, and 11T) exhibited lower acidity and reduced thickness compared to the control group. Post-fermentation freezing treatment effectively improved the perceived sourness and richness of the ice wine. The experiment proved that post-fermentation freezing treatment affected physicochemical indices, which in turn improved the acidity and thickness of ice wine and made the flavor of ice wine more harmonious. At the same time, it affected the differential expression of volatile compounds, which enriched the aroma of ice wine through different aroma intensities. This research provides a theoretical basis for improvement in the production process of ice wine.

## Figures and Tables

**Table 1:** Physicochemical parameters of grape juice.

Samples	Total Sugar (g/L)	Total Acid (g/L)	Tannin (g/L)	Total Phenols(g/L)	Anthocyanin (g/L)	pH
Grape juice	368.94	13.32	2.53	2.23	317.06	3.42

Note: Total phenols expressed as gallic acid equivalent, tannins as tannic acid equivalent.

**Table 2:** Sample number list.

Sample Code	Treatment Temperature (°C)	% Vol.
9C	16	9
10C		10
11C		11
9T	−20	9
10T		10
11T		11

**Table 3:** Sensory evaluation form.

Property	Percentage	Characteristics	Full Marks
Color	10%	Chroma and hue (color)	10
Clarity	10%	Level of clarity	10
Scent	30%	Subtlety	5
		Concentration	5
		Coordination	5
		Breed characteristics	5
		Duration	5
		Variety and complexity (multiple layers of aromas)	5
Taste	40%	Balance and harmony	10
		Body and concentration (sense of weight in the mouth)	10
		Sense of texture and structure	5
		Continuity and sense of layering	5
		Quality and persistence of chewing gum	5
		Aftertaste	5
Typicality	10%	Comprehensive review	10
Total score			100

**Table 4:** Organic acids of Beibinghong ice wine samples from different treatments after fermentation.

Sample	Tartaric Acid/g/L	Malic Acid/g/L	Lactic Acid/g/L	Glacial Acetic Acid/g/L	Citric Acid/g/L	Succinic Acid/g/L
9C	2.90 ± 0.04a	8.17 ± 0.03a	0.94 ± 0.14bc	3.24 ± 0.55b	0.75 ± 0.09a	1.97 ± 0.07a
9T	1.70 ± 0.12d	5.24 ± 0.32d	0.75 ± 0.25cd	1.64 ± 0.22c	0.54 ± 0.15bc	1.26 ± 0.36b
10C	2.17 ± 0b	8.12 ± 0.02a	1.11 ± 0.02ab	4.40 ± 0.17a	0.64 ± 0.02ab	1.97 ± 0.02a
10T	1.40 ± 0.02f	4.70 ± 0.01e	0.53 ± 0.01d	1.76 ± 0.1c	0.42 ± 0.01c	1.38 ± 0.05b
11C	1.99 ± 0.02c	7.81 ± 0.03b	1.19 ± 0.08a	4.02 ± 0.13a	0.68 ± 0.14ab	2.06 ± 0.18a
11T	1.60 ± 0.04e	5.73 ± 0.11c	0.62 ± 0.02d	2.11 ± 0.01c	0.46 ± 0.02c	1.27 ± 0.02b

Means with different letters in the same column express significant differences (Duncan’s test *p* < 0.05).

**Table 7:** Sensory evaluation scores.

Item	9C	10C	11C	9T	10T	11T
Color	9.68 ± 0.5	9.08 ± 0.7	9.41 ± 0.61	9.45 ± 0.35	9.54 ± 0.32	9.4 ± 0.38
Clarification	9.88 ± 0.16	9.67 ± 0.27	9.42 ± 0.43	9.82 ± 0.32	9.82 ± 0.33	9.46 ± 0.52
Aroma	24.61 ± 3.32	23.67 ± 3.09	23.85 ± 2.62	23.74 ± 1.93	27.5 ± 1.6	22.84 ± 1.77
Taste	33.93 ± 3.8	31.97 ± 2.22	34.19 ± 2.69	32.35 ± 2.66	34.71 ± 2.15	33.4 ± 2.06
Typicality	9.44 ± 0.43	9.1 ± 0.42	9.34 ± 0.36	9.54 ± 0.27	9.73 ± 0.33	9.19 ± 0.52
Totals	87.54 ± 3.63	83.48 ± 3.67	84.23 ± 8.48	84.9 ± 3.06	91.29 ± 2.89	84.29 ± 3.97

## Data Availability

The original contributions presented in the study are included in the article, further inquiries can be directed to the corresponding author.

## Supplementary Materials

The following supporting information can be downloaded at https://www.mdpi.com/article/10.3390/foods14091631/s1. Figure S1. Heat map of correlation clustering between taste indicators and physical and chemical indicators; Table S1. Standard curves for six organic acids; Table S2. Taste evaluation sheet; Table S3. Aroma evaluation sheet.
