# Supplementary material for: Analysis of the Effects of Post-Fermentation Freezing Treatment on the Flavor Characteristics of Beibinghong Ice Wine by HPLC and HS-GC-IMS
Source: Foods. 2025 May 6;14(9):1631. doi: 10.3390/foods14091631 (PMC12071324; doi:10.3390/foods14091631)
Supplement: Supplementary file 1 [file foods-14-01631-s001.zip › Supplementary Materials.pdf]

Table S1. Standard curves for six organic acids.

Compound	Calibration curve	R^2
Tartaric acid	f(x) = 2366.4x + 0.9989	1
Malic acid	f(x) = 1239.1x - 4.3401	0.9999
Lactic acid	f(x) = 1123.8x - 7.0687	0.9999
Glacial acetic acid	f(x) = 832.47x - 6.3193	1
Citric acid	f(x) = 1548.9x - 18.395	0.9998
Succinic acid	f(x) = 729.12x - 14.285	0.9999

Table S2. Taste Evaluation Sheet

Flavor	Full marks
Balance and harmony (when all the ingredients in a wine are in harmony so that none of them overshadow the others)	10
Thickness, ie the amount of consistency felt in the mouth	10
Astringency (a feeling of tightness or pucker in the mouth, tongue, or throat)	10
Aftertaste (the taste of wine left in the mouth after swallowing it)	10
Hierarchy (meaning different aromas and flavors in the front, middle and back of the wine, with layers)	10
Acidity	10

Table S3. Aroma Evaluation Sheet

The scent	Full marks
Floral	9
Fruity	9
Plant and Herbal	9
Fermented	9
Tarry	9
Honey	9

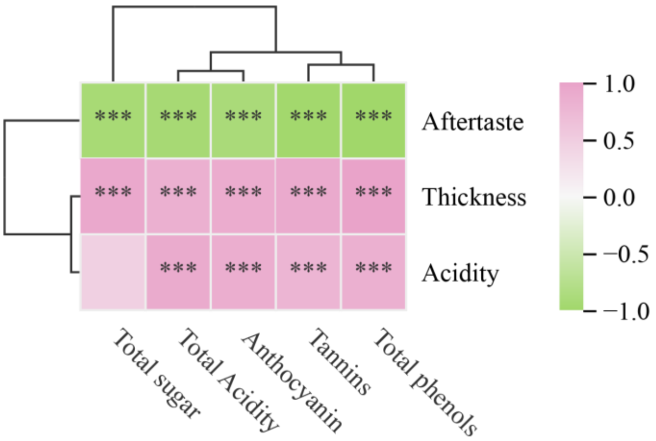

Figure S1. Heat map of correlation clustering between taste indicators and physical and chemical indicators.
